# Renal sympathetic denervation after Symplicity HTN-3 and therapeutic drug monitoring in severe hypertension

**DOI:** 10.3389/fphys.2015.00009

**Published:** 2015-02-09

**Authors:** Fadl Elmula M. Fadl Elmula, Anne C. Larstorp, Sverre E. Kjeldsen, Alexandre Persu, Yu Jin, Jan A. Staessen

**Affiliations:** ^1^Departments of Cardiology and Internal Medicine, Oslo University HospitalUllevaal, Norway; ^2^Faculty of Medicine, University of OsloOslo, Norway; ^3^Pole of Cardiovascular Research, Institut de Recherche Expérimentale et Clinique, Université Catholique de LouvainBrussels, Belgium; ^4^Division of Cardiology, Cliniques Universitaires Saint-Luc, Université Catholique de LouvainBrussels, Belgium; ^5^Studies Coordinating Centre, Research Unit Hypertension and Cardiovascular Epidemiology, KU Leuven Department of Cardiovascular Sciences, University of LeuvenLeuven, Belgium; ^6^VitaK Development and Research, Maastricht UniversityMaastricht, Netherlands

**Keywords:** hypertension, antihypertensive drugs, renal denervation, drug monitoring, treatment resistance

## Abstract

Renal sympathetic denervation (RDN) has been and is still proposed as a new treatment modality in patients with apparently treatment resistant hypertension (TRH), a condition defined as persistent blood pressure elevation despite prescription of at least 3 antihypertensive drugs including a diuretic. However, the large fall in blood pressure after RDN reported in the first randomized study, Symplicity HTN-2 and multiple observational studies has not been confirmed in five subsequent prospective randomized studies and may be largely explained by non-specific effects such as improvement of drug adherence in initially poorly adherent patients (the Hawthorne effect), placebo effect and regression to the mean. The overall blood-pressure lowering effect of RDN seems rather limited and the characteristics of true responders are largely unknown. Accordingly, RDN is not ready for clinical practice. In most patients with apparently TRH, drug monitoring and improvement of drug adherence may prove more effective and cost-beneficial to achieve blood pressure control. In the meantime, research should aim at identifying characteristics of those patients with truly TRH who may respond to RDN.

## Introduction

Renal sympathetic denervation (RDN) has been and is still proposed as a new treatment modality in patients with apparently treatment resistant hypertension (TRH), a condition defined as persistent blood pressure elevation despite prescription of at least 3 antihypertensive drugs including a diuretic (Krum et al., 2009, 2014; Esler et al., 2010). However, with the recent publication of the Symplicity HTN-3 study in the U.S. (Bhatt et al., 2014) it is questioned whether RDN at all lowers blood pressure (Demaria, 2014). During 2014, a total of 5 prospective and randomized studies of RDN showing modest or no effect on blood pressure in patients with TRH have been published or presented. Other recent studies have shown that patients with TRH have surprisingly low drug adherence. The aim of this paper is to review all prospective and randomized studies of RDN in TRH and, and to review the issue of poor drug adherence and suggest therapeutic drug monitoring (TDM) as a cost-effective modality to control blood pressure and improve prognosis in this subset of hypertensive patients who are at risk and difficult-to-treat.

## The rise and fall of renal denervation in treatment resistant hypertension

The initial enthusiasm followed by the setback of RDN can probably be summarized by a handful of explanations: (1) The role of the sympathetic system in the pathophysiology of hypertension is substantiated by a wealth of experimental and clinical arguments (Julius and Esler, 1975; Eide et al., 1979; Kjeldsen et al., 1981). On this background, enthusiasm surged when an intervention in this system seemed to drastically lower blood pressure. (2) Market-driven industry interests significantly influenced the medical community. (3) Subsequently, pitfalls in the treatment of apparent TRH patients, which are simple but well-known for decades, were suddenly forgotten, including well described phenomena such as the placebo effect, poor drug adherence (Gifford, 1988; Klein, 1988; Ceral et al., 2011) and the Hawthorne effect (Mangione-Smith et al., 2002). Regression to the mean could also be involved which means that abnormal BP values tend to change toward normalization without an underlying biological explanation.

The first and for a long time the only prospective randomized clinical trial in this field, the Symplicity HTN-2 study (Esler et al., 2010), was monitored by Ardian (Medtronic) who collected and processed the data. Usually, when such a task is given to industry, all measures are taken to secure confidence and trials are double-blinded (Julius et al., 2004). However, in this case, everything was open, making the trial particularly vulnerable to patient and physician related biases (Howard et al., 2013). In a recent editorial (Shun-Shin et al., 2014), the authors wrote that “measurement of a noisy variable by unblinded optimistic staff is a known recipe for calamitous exaggeration.” It is also unfortunate that selection of patients enrolled in Symplicity HTN-2 and evaluation of efficacy were based on office rather than ambulatory blood pressure measurements (ABPM), which is state-of-the art (O'Brien et al., 2013), particularly in resistant hypertension (Persu et al., 2014d). ABPM reduces observer bias and measurement error, minimizes the white-coat effect and has greater reproducibility, and therefore provides a better estimate of a patient's usual blood pressure and cardiovascular prognosis (Kikuya et al., 2007; Salles et al., 2008). Notwithstanding the well-known, major contribution of poor drug adherence to apparently resistant hypertension (Gifford, 1988; Klein, 1988; Ceral et al., 2011), drug adherence was not monitored, either at baseline or during follow-up. This made the study vulnerable to the Hawthorne effect, i.e., patients changing behavior—in this case starting taking their drugs as prescribed -, in response to the intervention and massive attention devoted to them. The lack of blood pressure decrease in the control group also raises concerns. One would indeed suspect that patients in the control group had not taken their medications properly, in order to keep their blood pressure at a higher level that made them eligible for cross-over to RDN group (Azizi et al., 2012; Persu et al., 2012). Finally, placebo effect and regression to the mean must also be taken into account. Noteworthy, the placebo effect is small by using ABPM (Staessen et al., 1996; O'Brien et al., 2013); however, ABPM remains as sensitive to the Hawthorne effect as office blood pressure.

## The role of industry in promoting renal denervation

Despite the major limitations and potential biases of Symplicity HTN-2, RDN was adopted in hundreds of centers worldwide. Medtronic Inc® (Minneapolis, Minnesota) paid $800 million to purchase Ardian® (Mountain View, California), the company that had developed the technology (Demaria, 2014), and more than 10 companies developed their own RDN systems, five of which obtained the CE mark (Conformité Européenne, European Conformity). CE marking means that the product is assessed before being placed on the market and meets EU safety, health and environmental protection requirements. However, CE marking is unrelated to medical indication at variance with the USA where FDA approves a medical device only when it has been tested and proved effective for a certain medical condition. The procedure was quickly reimbursed in Germany, and later on in Switzerland, Sweden and the Netherlands. While RDN remained an investigational procedure in the U.S., at least 8000 (Lüscher and Mahfoud, 2014), possibly 15,000–20,000 procedures were performed in Europe and in the rest of the world in less than 4 years, most of them using the Ardian -Medtronic® catheter. It may be hypothesized that the massive incomes, generated by selling the Symplicity catheter to enthusiastic Europeans paid for the Symplicity HTN-3 study (Bhatt et al., 2014), required by the FDA before approval of RDN in the U.S. In Symplicity HTN-3, blinding of patients through the use of a sham procedure and wider use of ABPM balanced and limited the differential impact of the Hawthorne, white coat, placebo and regression to the mean effects in both arms, disclosing to the world the true size of blood pressure decrease attributable to RDN, at least in patients meeting the Symplicity criteria; it was less than 2 mmHg systolic based on ABPM.

For all aforementioned reasons, and in view of the complexity and multifactorial character of hypertension, the failure of RDN to normalize or substantially reduce blood pressure in all patients with apparently TRH was a reasonable working hypothesis for us, even before the Medtronic announcement that Symplicity HTN-3 had failed to meet its primary endpoint (http://www.tctmd.com/show.aspx?id=123265). We (Fadl Elmula et al., 2013; Persu et al., 2013a,b) and others (Azizi et al., 2012; Howard et al., 2013) had predicted that the true effect of RDN might have been overestimated and may considerably shrink in properly designed studies (Howard et al., 2013), and that “one size may not fit all” (Persu et al., 2012). In particular, in preliminary analysis of the European Network COordinating research on Renal Denervation (ENCOReD) network (Persu et al., 2014a) we were struck by the imbalance between the 17.6 mmHg decreases in office blood pressure, vs. only 5.9 mmHg for 24-h ambulatory blood pressure.

## Finding patients with true treatment-resistant hypertension for research

**Figure 1:**
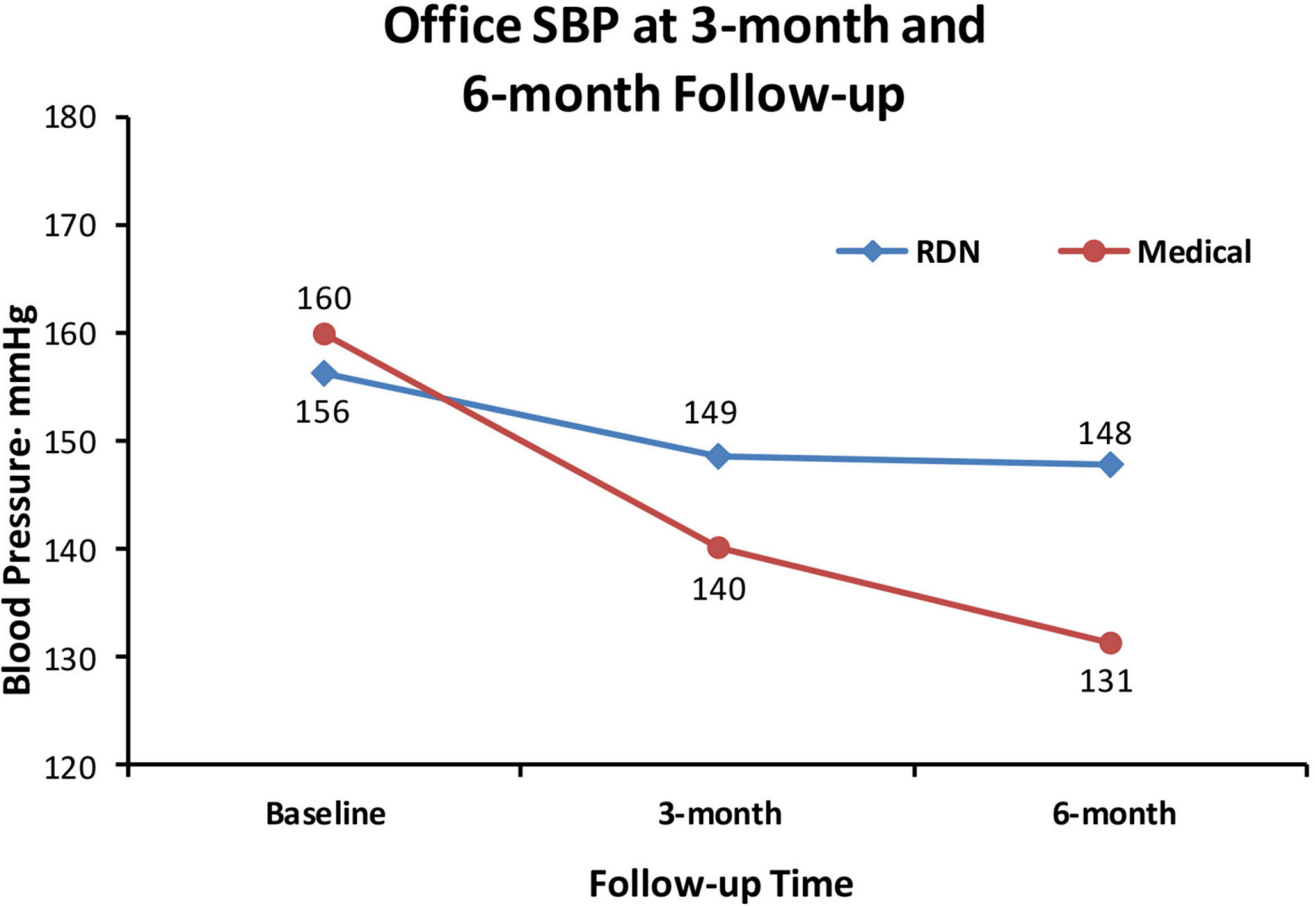
**Shows the effect of RDN on office systolic blood pressure (SBP) at 3-month and 6-months of follow-up, compared to drug treatment adjustment guided by non-invasive hemodynamic measurements**. Differences were statistically significant (Fadl Elmula et al., 2014), favoring drug treatment adjustment, which is the recommended method to gain blood pressure control in patients with so-called treatment-resistant hypertension (Gifford, 1988).

**Figure 2:**
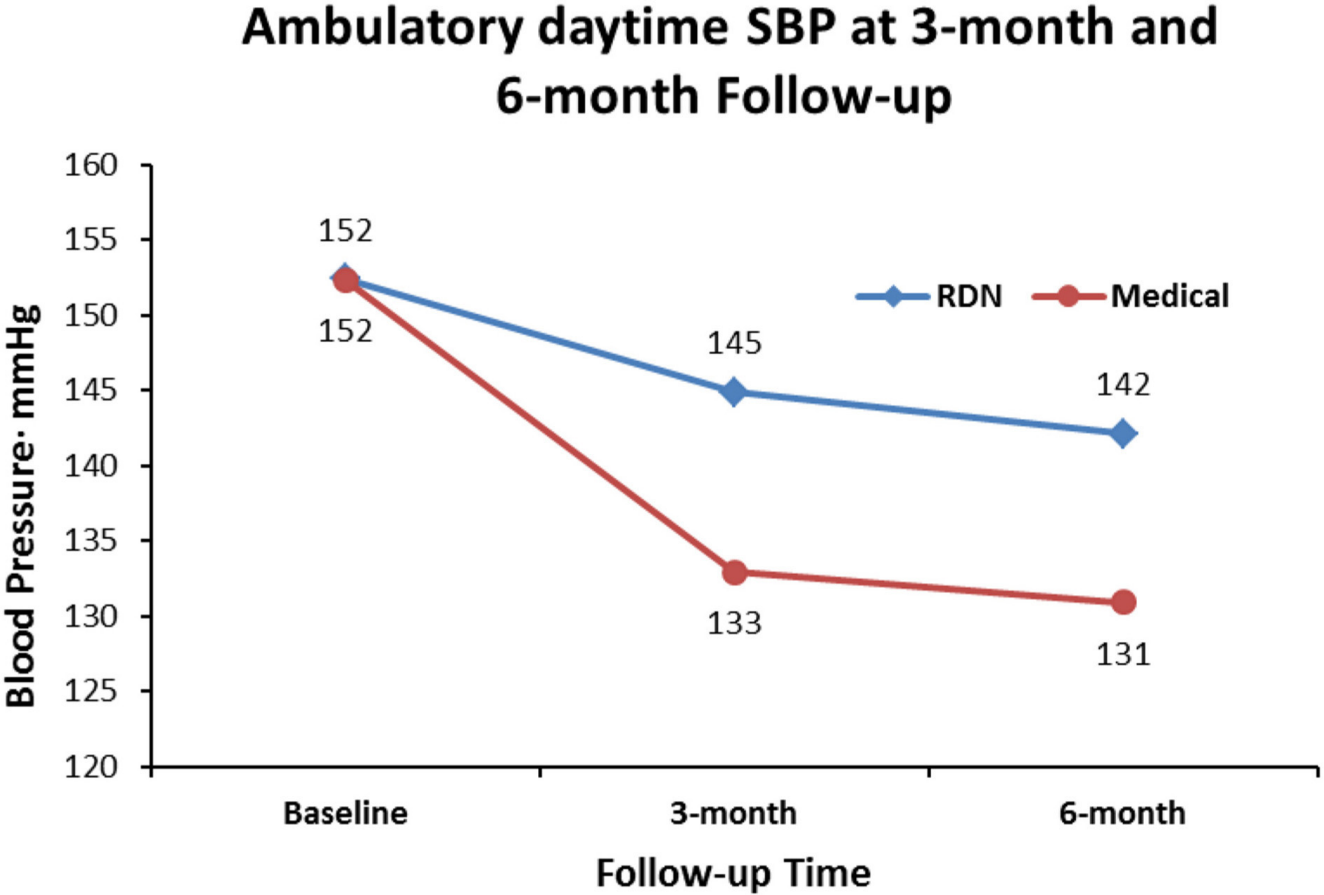
**Shows the effect of RDN on ambulatory daytime systolic blood pressure (SBP) at 3-month and 6-months of follow-up, compared to drug treatment adjustment guided by non-invasive hemodynamic measurements**. Differences were statistically significant (Fadl Elmula et al., 2014), favoring drug treatment adjustment, which is the recommended method to gain blood pressure control in patients with so-called treatment resistant hypertension (Gifford, 1988).

When we set out to investigate the effects of RDN in one of the centers with the longest experience in conducting randomized clinical trials in Europe (Helgeland, 1980), we had thus clearly in mind the limitations of previous studies. We needed a simple and practical way to deal with pitfalls in the recruitment of patients with resistant hypertension into a study protocol: Patients had to qualify for the RDN protocols by having elevated daytime ABPM after witnessed intake of their prescribed blood pressure medication (Fadl Elmula et al., 2013). Meanwhile, a leading hypertension center in Germany (Brinkmann et al., 2012) published a well-documented series of patients whose blood pressure remained unchanged after RDN. We were thus not surprised when we found no change in either office or ABPM following RDN, first in an open series of six patients (Fadl Elmula et al., 2013), later followed by a randomized study (Fadl Elmula et al., 2014). Patients who were randomly assigned to further improvement of drug treatment guided by non-invasive hemodynamic monitoring had normalized blood pressures (Figures 1, 2). In contrast, patients exposed to RDN experienced only a small and probably partly placebo-induced fall in office and ABPM. The decreases averaged 20 mmHg more for office and 9 mmHg more for ambulatory systolic blood pressure in the hemodynamically guided drug treatment group (*n* = 10) compared to the RDN group (*n* = 9). Because of sustained elevation of AMBP in the RDN treated patients at 6 months of follow-up, we stopped randomization for ethical reason according to a pre-specified decision (Fadl Elmula et al., 2013).

## The pitfalls with renal denervation in treatment resistant hypertension

In the absence of solid evidence of efficacy, how can we explain the uncontrolled deployment of RDN in Europe and worldwide (with the notable exception of the U.S. where RDN remained an investigational procedure)? Of course, publications of the Symplicity studies and multiple observational studies, and enthusiastic editorials and reviews in top-ranking journals (Mahfoud et al., 2013; Ott et al., 2013) had a substantial impact, and the lack of strict rules for introduction of device-based therapies in Europe facilitated the large-scale implementation of the technique. However, this phenomenon would have remained limited without the huge promotion by device-producing industry. Probably industry has never launched such a strong campaign to market a new technology before. A multitude of national and international advisory boards organized educational meetings, developed a website (www.poweroverpressure.com) and produced guidelines, and corresponding author of this current review contributed to these. Medical journals were swamped by reviews and meta-analyses showing the powerful blood pressure lowering effects as recorded in observational studies and in the single available randomized study, Symplicity HTN-2. Comments pointing out the defects and inconsistencies in such meta-analysis encountered great delay in getting published (Jin et al., 2014). Many never questioned *whether* RDN should be implemented, but *when* it should start in an institution. By all means, the purpose was to disseminate the enthusiasm for RDN from the technically-oriented invasive radiologists and cardiologists who usually had little interest or experience in the treatment of hypertension to the “hypertension establishment.” The European Society of Hypertension issued specific guidelines (Schmieder et al., 2012, 2013), but maintained reservations that more data was needed, and eventually it had to be proven that RDN would lower morbidity and mortality before being generally accepted in the treatment of true or apparent TRH.

In the aftermath of Symplicity HTN-3, it has been suggested that the lack of demonstrated efficacy of RDN in Symplicity HTN-3 may be due to lack of statistical power or even to chance (Lüscher and Mahfoud, 2014) or that the trial was well conceived but not rigorously executed (Esler, 2014; Schmieder, 2014; White et al., 2014; Lobo et al., 2015). In particular, a fraction of African American participants increased their antihypertensive medication, contrary to protocol, which masked a potential BP lowering effect of RDN in contrast to other participants. In addition, legitimate concerns were raised as to whether the denervation procedure was sub-optimal in many cases due to insufficient delivery of appropriate energy in the renal arteries as a consequence of the inexperience of the investigators. However, this criticism is all *post-hoc*, and the Symplicity HTN-3 findings are after all in line with the other RCTs published and presented in 2014 (Azizi et al., 2014; Desch et al., 2014; Fadl Elmula et al., 2014; Rosa et al., 2015). Furthermore, the Symplicity HTN-3 results are diluted by non-scientific comparisons with the Medtronic® registry (Pathak et al., 2014) which is hampered by all the weaknesses touched upon in this review, and even more as it is a pure industry-ran activity. Finally, while RDN will not become available in the U.S., and ongoing research in Asia was stopped, industry continues to make their catheters available for clinical use and promotes the technique in Europe.

## Could there be responders to renal denervation in hypertension?

Does the failure of Symplicity HTN-3 mean the end of RDN? Not necessarily. Indeed, it has been shown in cohorts recruited from the third (The effect of progressive sympathectomy on blood pressure, Bradford Cannon, 1931) until the fifth decade of the last century (Smithwick and Thompson, 1953; Longland and Gibb, 1954) that abdominal sympathectomy associated to splanchnicectomy is effective in the treatment of severe hypertension. Accordingly, research should go on to find the minority of patients who are true responders to RDN, and identify predictors of effective RDN. The European Network COordinating research on Renal Denervation (ENCOReD) is set up to include thousands of patients in randomized protocols, observational studies and registries independent of industry. Some early results (Persu et al., 2014a,b) from this joint effort have already been published and suggest that it may be worthwhile searching for potential predictors of response to RDN. When 2 prospective and randomized studies that have been published (Rosa et al., 2015) and reported (Azizi et al., 2014), plus Symplicity Flex, another sham-controlled study very recently reported (Desch et al., 2014) are added to the 3 published studies (Esler et al., 2010; Bhatt et al., 2014; Fadl Elmula et al., 2014) the overall picture shows that RDN is equal to drugs in lowering BP. However, individual data suggests that there may be cause for optimism that some truly responding patients may be identified.

Still, before going ahead, we have to draw the lessons of the RDN story. We must make sure that RDN is beneficial and does no harm. Many patients have probably undergone unneeded procedures. By a careful estimate, 20 000 renal arteries have been exposed to ablation in people with hypertension and an increasing number of cases of renal artery stenosis after RDN are being reported (Persu et al., 2014c). It remains to be seen whether the negative news that RDN is not for most people will reach Time Magazine (Oz, 2012) and Der Spiegel (Blech, 2013), or whether the old lessons (Bramley et al., 2006) remain for clinicians who treat people with hypertension in daily life.

## Therapeutic drug monitoring in resistant hypertension

In view of the major contribution of poor drug adherence to apparent TRH, therapeutic drug monitoring (TDM) maybe a useful tool for detecting and reducing non-adherence, leading to substantial blood pressure (BP) improvement in this subset of hypertensive patients. (Chung et al., 2014) have assessed cost-effectiveness of TDM using a Markov model based on German data and life statistics to evaluate life-years, quality-adjusted life-years (QALYs), costs, and incremental cost-effectiveness ratios in TRH patients receiving either TDM optimized therapy or standard best medical therapy. Efficacy of TDM was modeled by reducing risk of hypertension-related morbidity and mortality. The authors showed that TDM is a cost-effective health care intervention in patients diagnosed with TRH, and that this finding is valid for a wide range of patients, irrespective of age and sex (Chung et al., 2014).

Poor drug adherence in apparent TRH is a serious issue that has drawn the attention of experienced clinicians for many years (Gifford, 1988; Klein, 1988). Recently, in a study of 84 patients taking on average 5 antihypertensive drugs it was shown by measurements that no drug was detectable in the blood in 34.5% of the patients, and 65.5% of the patients fulfilled the criteria of non-adherence (Ceral et al., 2011). Other investigators have provided similar results (Jung et al., 2013; Strauch et al., 2013; Tomaszewski et al., 2014). Beyond the clinical challenge of convincing people with severe hypertension to take their antihypertensive medication in order to control their high blood pressure and improve their prognosis, changes in drug adherence over time may have major, unpredictable effects on the results of clinical trials including patients with apparent TRH. People may change their behavior when given special attention in research (the Hawthorne-effect). This may introduce important biases, as patients with assumed TRH but with poor drug adherence, may start taking their drugs when exposed to additional intervention. We postulate that much of the recent controversy with RDN can be explained in this way (Esler et al., 2010; Bhatt et al., 2014).

Clinical assessment of non-adherence in routine practice is challenging (Burnier et al., 2003). Drug adherence is usually investigated using written patient's diary or somewhat more sophisticated by electronic pill boxes, or blood and urine measurements of prescribed drugs. Measurements of drugs can provide interesting information, but are not often used in practical clinical work especially in primary care, and the cost has been prohibitive until recently. Neither patient's diary nor electronic pill boxes are perfectly reliable to ensure drug intake. The only methods that 100% ensures true drug intake is witnessed drug intake, an approach that may yield quite interesting results in patients with TRH (Fadl Elmula et al., 2013, 2014). However, while witnessed intake of drugs may identify adherent patients for immediate inclusion into a study, this method is not particularly practical in the long-run for the follow-up in clinical practice or research.

In the long run, TDM in body fluids may thus prove the best tool for evaluation and improvement of adherence to drug therapy (Brinker et al., 2014). This approach allows an objective surveillance of patient adherence by repeatedly measuring concentrations of antihypertensive drugs in blood and urine. Moreover, when non-adherent patients were confronted with their low or undetectable drug levels and were provided additional counseling to overcome barriers of adherence, blood pressure control improved considerably without intensification of therapy (Brinker et al., 2014). While several studies as pointed out above focused on the objective exclusion or confirmation of non-adherence, this recent study (Brinker et al., 2014) utilized the information gained from TDM measurements for therapeutic purposes. The TDM results were discussed with the non-adherent patients to explore barriers to adherence and counseling was provided to overcome these barriers. During follow-up, SBP was reduced by 46 ± 10 mmHg in non-adherent compared to 12 ± 17 mmHg in adherent patients, without intensification of the antihypertensive therapy (Brinker et al., 2014).

TDM identifies and helps to resolve the key problem in many—possibly the majority—of patients with apparent TRH—that is poor adherence to prescribed drug regimen. As previously shown, the cost-effectiveness of this approach is supported by a solid rationale (Chung et al., 2014) and should not be compared to similar analyses of controversial device intervention (Geisler et al., 2012; Dorenkamp et al., 2013; Gladwell et al., 2014) in apparent TRH patients. So far, such analyses were indeed based on Symplicity HTN-2, an unblinded study largely open to the Hawthorne and placebo effects, whose results could not be replicated in any of five randomized trials published or presented in 2014.

### Conflict of interest statement

Sverre E. Kjeldsen, Fadl Elmula M. Fadl Elmula and Anne C. Larstorp have been supported by grant from Hemo Sapiens; and Sverre E. Kjeldsen by unrestricted grants from AZ and Pronova. Sverre E. Kjeldsen has received lecture and consultancy honoraria from AZ, Bayer, Medtronic, MSD, Novartis, Serodus, and Takeda, and royalty payments from Gyldendal (publisher). Fadl Elmula M. Fadl Elmula has received speaker honorarium from Medtronic and Hemo Sapiens and Anne C. Larstorp from Hemo Sapiens and Merck et Co. Alexandre Persu declares no conflict of interest.

## Abbreviations

ABPM: ambulatory blood pressure monitoring
RDN: renal sympathetic denervation
TRH: treatment resistant hypertension
TDM: therapeutic drug monitoring.
